# Heterochronic limb patterning in marsupials reveals flexibility in the processes underlying lateral plate mesoderm morphogenesis

**DOI:** 10.1126/sciadv.aed3192

**Published:** 2026-07-15

**Authors:** Axel H. Newton, Alexandra Leggatt, Ella R. Farley, Karen E. Sears, Aidan M. Couzens, Sara Ord, Andrew J. Pask

**Affiliations:** ^1^ The School of BioSciences, University of Melbourne, Victoria, 3010, Australia.; ^2^ Department of Ecology and Evolutionary Biology, University of California, Los Angeles, CA 90095, USA.; ^3^ College of Science & Engineering, Flinders University, Adelaide, 5042, Australia.; ^4^ Colossal BioSciences, Dallas, TX 75247, USA.

## Abstract

Marsupial embryos develop under intense functional constraints: neonates are born after a short gestation but must immediately crawl to the teat using precociously developed forelimbs. To meet this demand, marsupials have evolved extreme acceleration of limb morphogenesis, yet the cellular and molecular mechanisms underlying this shift remain unresolved. Using two distantly related marsupials, the fat-tailed dunnart (*Sminthopsis crassicaudata*) and the gray short-tailed opossum (*Monodelphis domestica*), we show that this acceleration extends upstream to the earliest stages of lateral plate mesoderm (LPM) formation. The forelimb field is specified in relative isolation from other axial structures, marked by accelerated activation of limb genes prior to neural tube and somite formation. Forelimb outgrowth begins before overt LPM subdivision and epithelial–mesenchymal transitions, with limb mesenchyme arising from an undifferentiated LPM. These findings reveal unexpected flexibility in the temporal relationships between axial morphogenesis and limb initiation, highlighting evolutionary plasticity in the processes that govern vertebrate limb patterning.

## Teaser

Accelerated limb formation in marsupials reveals canonical developmental landmarks aren’t strict prerequisites for initiation.

## INTRODUCTION

The tetrapod limb – from fins to wings and hands with opposable digits – has long served as a model for understanding how complex tissues are patterned, built, and diversified during development and evolution. Work in established model systems, such as the chicken and mouse, has defined the core gene regulatory networks that drive limb development [for reviews see (*1*–*3*)], while comparative studies across non-model taxa have revealed how modulation of these programs generates morphological diversity (*4*–*10*). In these systems, limb formation is understood to proceed through a conserved sequence of morphogenetic events originating in the lateral plate mesoderm (LPM) (*11*). Following mediolateral segmentation of the mesoderm into axial, paraxial, and lateral domains (*12*–*15*), the LPM undergoes epithelialisation and dorsoventral subdivision into somatic and splanchnic layers, separated by the coelomic cavity (*16*, *17*). This transformation establishes distinct developmental fates, whereby the somatic LPM contributes to formation of the body wall and limbs, and the splanchnic LPM gives rise to the visceral organs. In tetrapod models such as the mouse and chicken, forelimb induction is closely associated with this subdivision, spatially segregating the somatic LPM into a distinct tissue layer allowing restricted activation of the limb gene regulatory network (*16*, *18*, *19*), followed by a localized epithelial-to-mesenchymal transition (EMT) to generate the early limb bud mesenchyme (*20*). This ordered progression of segmentation, subdivision, and outgrowth has come to define the canonical trajectory of limb development.

Despite substantial progress in defining the molecular pathways of limb development, the earliest morphogenetic events that lead to establishment of the limb fields are less well resolved (*21*, *22*). Within the presumptive forelimb domain but prior to limb initiation, the early somatic LPM exhibits progressive activation of transcription factors such as PRRX1, IRX3 and TWIST1 (*16*, *23*–*27*), although these appear to be individually dispensable for limb development (*28*, *29*). Forelimb initiation is instead dependent on targeted activation of the transcription factor TBX5 in the somatic LPM (*30*–*32*), which in turn triggers an FGF10–FGF8 feedback loop to sustain limb outgrowth (*33*–*35*). Spatially restricted *Tbx5* expression is thought to arise through a complex interplay of upstream cues, including colinear *Hox* gene expression (*36*–*39*) and WNT (*32*, *39*, *40*), BMP (*16*, *41*) and retinoic acid (RA) (*42*–*44*) signaling pathways, although the extent to which these inputs act cooperatively or independently remains unresolved (*39*, *43*). The subdivision of the LPM is assumed to help spatially organize these signals within the limb-forming domains (*16*). However, resolving the source and temporal hierarchy of these inductive cues is complicated by the concurrent development of neighbouring axial structures, including signaling by the ectoderm, neural tube, notochord, somites, and intermediate mesoderm (*18*). Moreover, the tight coupling of axial and limb development in conventional models such as chicken and mouse makes it difficult to disentangle the relative contributions of these tissues to forelimb induction.

**Fig. 1.**
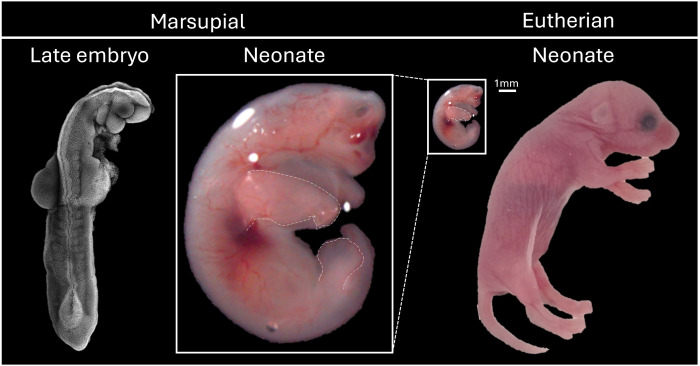
Accelerated forelimb development in marsupials. Marsupial dunnart (*Sminthopsis crassicaudata*) embryo and neonate showing accelerated forelimb development. The altricial neonates possess robust forelimbs to crawl to the teat yet are considerably smaller and less developed than mouse neonates.

Marsupial mammals provide a powerful system to address these challenges owing to their distinctive developmental mode. Unlike eutherian mammals, which form limbs in parallel with surrounding embryonic structures, marsupials initiate forelimb development in relative physical and temporal separation from other axial tissues (*45*). Marsupial neonates are born at highly altricial stages after a short gestation, yet must use their forelimbs to crawl to the teat immediately after birth – a functional constraint that has driven the evolution of accelerated (heterochronic) forelimb development (Fig. 1) (*46*, *47*). This includes precocious expression of canonical limb regulators, such as *TBX5, FGF10, FGF8, SHH* (*48*), and an expanded forelimb field spanning more somites than mouse or chicken (*45*, *49*–*51*). Notably, visible forelimb buds emerge during early stages of axial development, coinciding with or preceding the formation of structures such as somites and the neural tube (*48*). These features suggest that the processes underlying forelimb specification and initiation may occur under an accelerated timeline compared with canonical morphogenetic events, making marsupials a valuable system to interrogate the earliest stages of limb field formation in a comparatively simplified developmental context.

In this study, we used two distantly related marsupial models, the fat-tailed dunnart (*Sminthopsis crassicaudata*) (*52*) and the gray short-tailed opossum (*Monodelphis domestica*) (*45*) representing two distinct superorders diverging approximately 80 million years ago (*53*), to dissect the cellular and molecular mechanisms underlying LPM formation and limb field specification. By visualizing key molecular markers of LPM specification and limb initiation, we traced the sequence of morphogenetic events accompanying the heterochronic growth patterns characteristic of marsupial development. Our results show that forelimb is patterned prior to the formation of other axial structures, such as the neural tube and somites, and that early limb outgrowth occurs before overt subdivision of the LPM or establishment of a coelomic epithelium. These findings provide previously unexplored perspectives on the temporal relationships between morphogenetic processes and limb initiation. By examining these precocious developmental dynamics in marsupials, we reveal an unexpected degree of flexibility in the canonical programs governing early limb development.

## RESULTS

To characterise the onset of limb formation across marsupials, we compared the Australian marsupial *S. crassicaudata* (fat-tailed dunnart) (*52*) and the American *M. domestica* (gray short-tailed opossum) (*54*). Using whole-mount fluorescent RNA and protein imaging of key LPM and limb transcription factors, *PRRX1*, *FOXF1, TWIST1*, and *TBX5*, we defined the temporal and spatial onset of LPM specification in early marsupial embryos. *PRRX1* is among the earliest marker of limb specification, with expression in the undifferentiated LPM that persists throughout the developing limb (*23*, *55*–*57*). *FOXF1* is similarly expressed in the early LPM but later becomes restricted to the splanchnic layer (*16*, *27*). Following LPM subdivision, *TWIST1* is specifically activated in the somatic LPM prior to *TBX5,* and persists throughout the limb mesenchyme (*18*, *25*, *26*), whereas *TBX5* serves as the definitive marker of forelimb initiation (*30*, *31*, *58*, *59*). Through visualization of these markers, we found that marsupial LPM specification and limb initiation occur rapidly over three developmental stages (stages 20–22, ~8 hours), representing a marked heterochronic shift compared to mouse and chicken development (*18*, *20*).

### Limb field specification

**Fig. 2.**
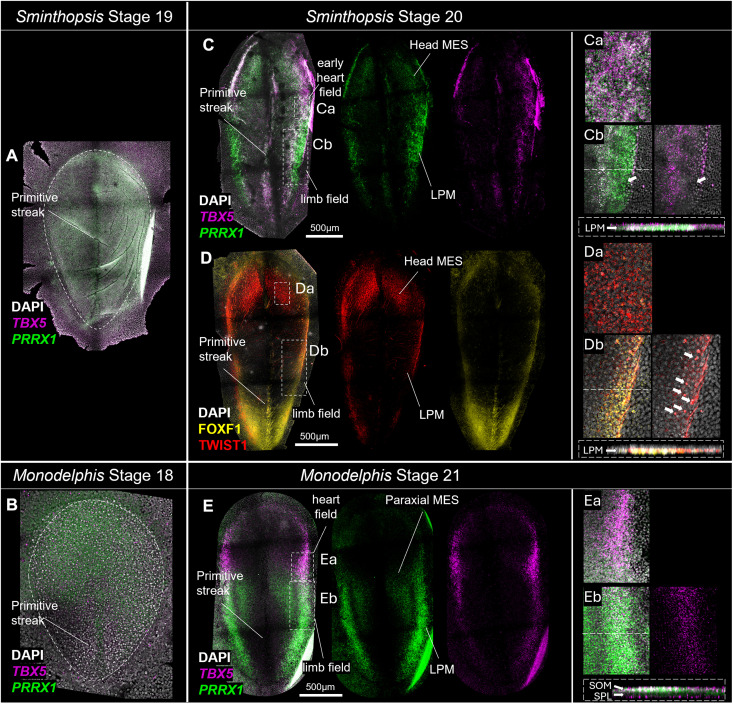
LPM specification in marsupial embryos. (**A**) Stage 19 *Sminthopsis* and (**B**) stage 18 *Monodelphis* embryos showing lack of bilateral LPM domains, through *PRRX1 or TBX5* expression*.* (**C**) Stage 20 *Sminthopsis* embryo showing formation of bilateral LPM domains via positive expression of *PRRX1* in the LPM, and co-expression with TBX5 in the heart field (Ca), but no compelling overlapping expression in the presumptive limb field (Cb), suggesting limb induction has not yet begun. (**D**) Stage 20 *Sminthopsis* embryo, marked by TWIST1 and FOXF1 demonstrating the somatic LPM has yet to form. Robust TWIST1 expression is seen in the head mesoderm (Da), and some positive nuclei can be observed in the limb field (Db), suggesting somatic LPM specification may be beginning, though optical sections reveal the LPM is still unilaminar. (**E**) Stage 21 *Monodelphis* embryo showing co-expression of *PRRX1* and *TBX5* in both the heart (Ea) and limb (Eb) field confirming limb induction. Optical sections reveal the bilaminar LPM, with both *PRRX1* and *TBX5* restricted to the dorsal somatic layer. LPM, lateral plate mesoderm; som, somatic LPM; spl, splanchnic LPM.

Marsupial organogenesis begins at McCrady stage 18-19 with formation of primitive streak in the tear-drop shaped embryonic disc (*52*, *54*). Fluorescent imaging of *PRRX1* and *TBX5* RNA in *Sminthopsis* and *Monodelphis* embryos did not detect expression at these stages, suggesting the lateral plate mesoderm had not yet become segmented from the primitive mesoderm (Fig. 2, A and B). Shortly thereafter, at stages 20-21 as the embryonic disc elongates, labelling of *PRRX1* RNA and FOXF1 protein are detected in bilateral lateral domains, consistent with LPM specification (Fig. 2, C and D). At this stage forelimb initiation had not yet begun: *TBX5* expression was restricted to the heart field, with no clear overlap with *PRRX1* in the presumptive forelimb field (Fig. 2C). Similarly, TWIST1 protein was robust in the head mesoderm but not clearly detected in the forelimb field, although occasional positive nuclei were present suggesting early onset of somatic LPM specification [Fig. 2D (b)].

Equivalent *PRRX1* and *TBX5* labelling in *Monodelphis* revealed similar patterns, with distinct separation between the *TBX5*-positive cardiac domain and *PRRX1*-positive LPM, alongside a small region of overlap in the presumptive forelimb field (Fig. 2E). Notably, somites had not yet formed at this stage. Optical z-sections further showed that *PRRX1* was confined to a single LPM layer in stage 20 *Sminthopsis* embryos [Fig. 2C (b)], whereas in stage 21 *Monodelphis*, *PRRX1* and *TBX5* were co-expressed within a dorsally restricted somatic LPM layer [Fig. 2E (b)]. Together, these observations indicate that forelimb fields are specified rapidly within the LPM, prior to the formation of key axial structures, including somites and the closed neural tube.

### Transition to limb outgrowth

**Fig. 3.**
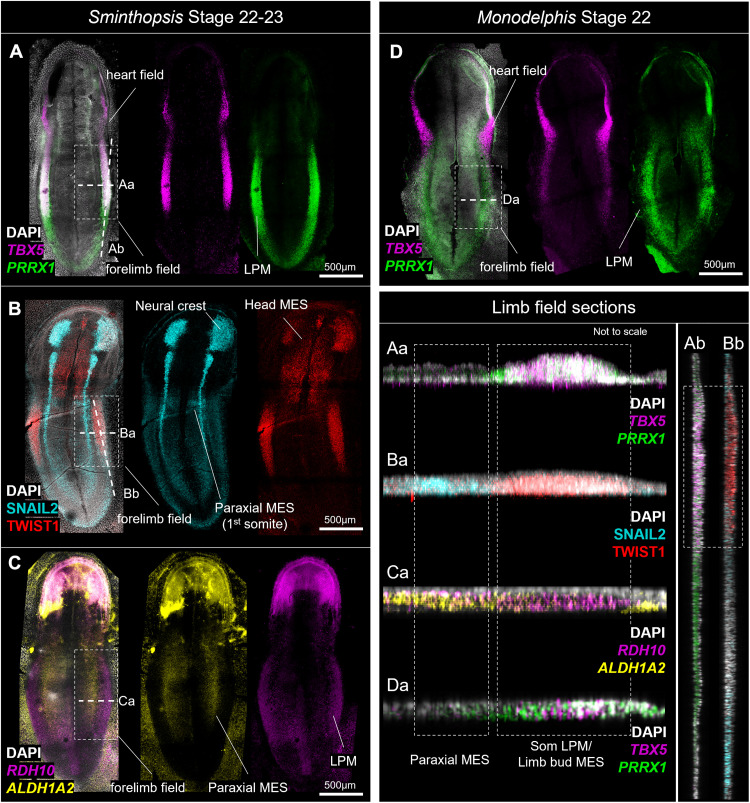
Onset of limb induction in marsupial embryos. Stage 22-23 *Sminthopsis* and *Monodelphis* embryos stained for key limb field specification markers. Sminthopsis embryos showing formation of distinct forelimb fields observed through co-localization of *PRRX1* and *TBX5* RNA (**A**), and TWIST1 and SNAI2 protein (**B**). The early limb buds can already be seen protruding from the flat embryo, whilst the SNAI2 paraxial mesoderm at the level of the limb field is rudimentary [(A) a and b, and (B), a and b]. The first SNAI2 positive condensing somite pair can be seen anterior to the limb field (B). (**C**) Expression of retinoic acid synthesis enzyme genes *RDH10 & ALDH1A2* in the *Sminthopsis* limb fields show complementary domains with RDH10 in the LPM and ALDH1A2 in the neighbouring paraxial mesoderm and extraembryonic mesoderm [(C) a]. (**D**) PRRX1 and TBX5 expression in stage 22 Monodelphis embryos showing similar patterns to *Sminthopsis*, with discrete expression of *TBX5* in the *PRRX1*-positive forelimb field [(D) a].

This rapid inductive timeline becomes more apparent in subsequent stages, where distinct forelimb buds begin to protrude from the flattened embryonic disc (*45*, *52*). Imaging of *PRRX1 and TBX5* RNA expression at stages 22-23 in both species revealed overlapping expression domains within forelimb fields, consistent with limb induction (Fig. 3, A and D). To assess somatic LPM specification, immunostaining for TWIST1 and SNAI2 in stage 22 *Sminthopsis* embryos identified a TWIST1-positive domain within the forelimb field, corresponding to somatic LPM-derived forelimb mesenchyme. This domain was clearly distinct from surrounding SNAI2-positive neural crest and paraxial mesoderm (Fig. 3B). At this stage, condensation of the first SNAI2-positive somite could be detected anterior to the forelimb field, while hindlimb fields were not yet evident (Fig. 3B).

Given the limited development of paraxial mesoderm at these stages, we next examined potential inductive signals contributing to early limb field specification. Retinoic acid (RA) signaling is a well-established upstream regulator of *TBX5* activation across vertebrates, making it a strong candidate pathway to examine in the context of heterochronic limb induction. RNA imaging of the RA synthesis enzymes *RDH10* and *ALDH1A2* revealed complementary spatial expression domains: *RDH10* was expressed within the LPM of the limb field, whereas *ALDH1A2* was detected in adjacent paraxial and extra-embryonic mesoderm [Fig. 3, (A) a to (D) a, and (C)], suggesting RA synthesis may be active at these stages. Notably, at these stages, the forelimb field already exhibited early mesenchymal thickening, while the rest of the embryo remained largely flat and only a few cells thick, with no clear morphological evidence of splanchnic LPM (Fig. 3D). These observations raise the possibility that limb initiation occurs prior to overt LPM subdivision, which typically precedes limb outgrowth in established amniote models (*16*, *20*).

### Subdivision of the lateral plate mesoderm occurs after forelimb initiation and outgrowth

**Fig. 4.**
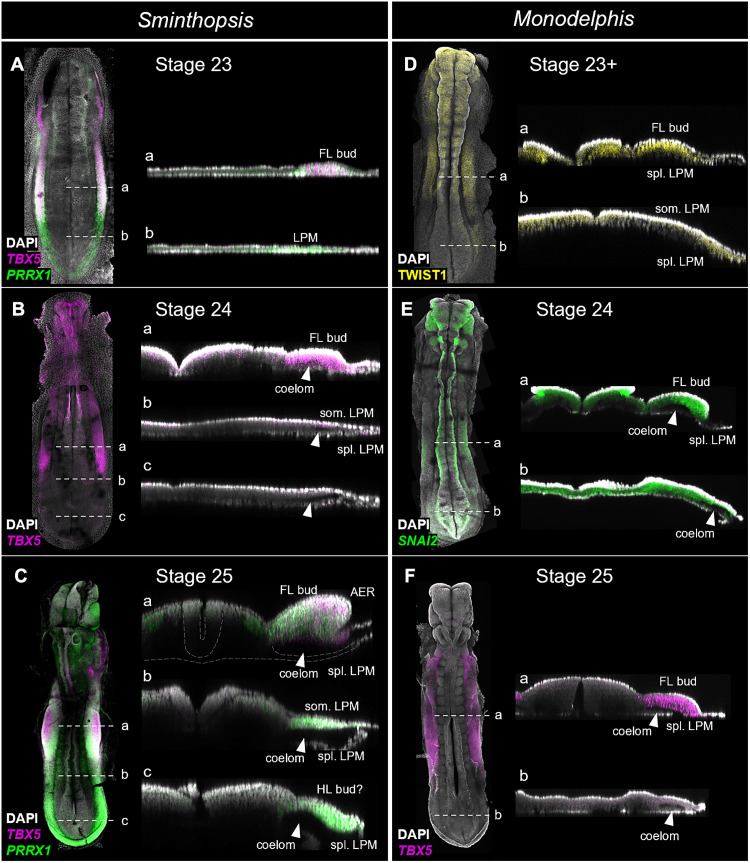
Timeline of LPM subdivision in marsupial embryos. Whole mount immunostaining of LPM/limb markers in *Sminthopsis* and *Monodelphis* embryos, with optical z-sections through the developing LPM. (**A** and **D**) At the time of early limb bud outgrowth (stage 23), no physical subdivision via presence of coelomic cavity can be observed at the level of the forelimb field [(A), a and (D), a] or posterior axial levels of the embryo. (**B**, **C**, **E**, and **F**) Shortly after at limb bud stages (stage 24 and 25), physical LPM separation via presence of the coelom can be seen at all axial levels, including that of the forelimb. These data suggest limb outgrowth precedes subdivision of the LPM. Panel (A) reused from Fig. 3.

Given the early appearance of limb bud mesenchyme, we next examined the timing of LPM subdivision relative to forelimb outgrowth. Whole-mount immunostaining and optical sectioning across successive stages of development revealed that at stage 23, when the forelimb bud is already visible, the LPM remains physically continuous with no detectable coelomic cavity separating somatic and splanchnic layers (Fig. 4, A and D). Shortly thereafter, at stages 24-25, separation of these layers becomes evident, with formation of a distinct coelomic cavity visible along the length of the embryo (Fig. 4, B, C, E, and F), including at the level of the presumptive hindlimb field. These observations indicate that LPM subdivision occurs prior to hindlimb initiation, but follows the onset of forelimb outgrowth (*45*).

**Fig. 5.**
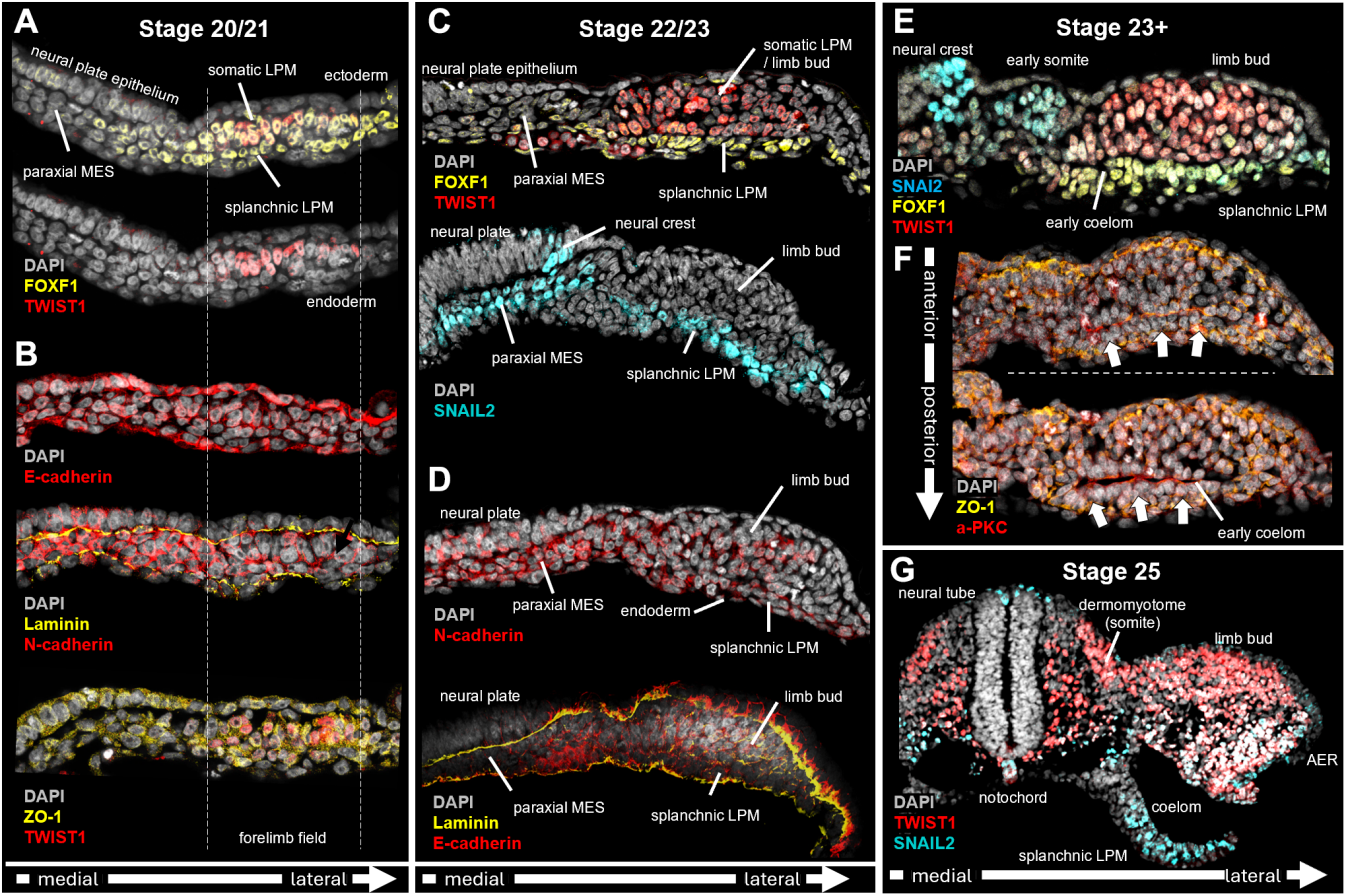
Molecular subdivision precedes physical subdivision of the lateral plate mesoderm during forelimb outgrowth. Tissue sections through the forelimb field at the onset of specification [stages 20/21; (A) and (B)], initiation [stages 22/23; (C) and (D)], early outgrowth [stage 23+; (E) and (F)] and established limb bud [stage 25; (G)] show that limb mesenchyme forms prior to physical separation of the LPM. TWIST1-positive somatic LPM and FOXF1-positive splanchnic LPM domains are molecularly segregated but remain physically continuous during early stages. At stages 20/21, the TWIST1-positive somatic LPM first emerges as a small domain within the broader FOXF1-positive LPM, remaining contiguous with the underlying splanchnic layer (**A**). Cells exhibit a disordered, mesenchymal-like morphology, with low and heterogeneous E-cadherin and N-cadherin localisation and no clear ZO-1-positive apical polarity (**B**). By stages 22/23, the TWIST1-positive domain expands, consistent with early limb mesenchyme, while remaining physically continuous with the FOXF1/SNAI2-positive splanchnic LPM (**C**). Despite dorsoventral molecular segregation, no coelomic cavity or clear evidence of apical polarity is present at this stage (**D**). At late stage 23, physical separation of the somatic and splanchnic LPM becomes apparent with the emergence of a coelomic cavity (**E**). This is accompanied by progressive epithelial organisation, including aPKC- and ZO-1-positive apical polarity along the forming coelomic boundary, marking early coelom formation (**F**). By stage 25, the coelomic cavity is clearly established between the expanded limb bud and underlying splanchnic LPM (**G**). LPM, lateral plate mesoderm; MES, mesoderm; AER, apical ectodermal ridge.

As optical sections suggested that the forelimb mesenchyme emerges prior to LPM subdivision, we next examined tissue sections from stage 20–25 dunnart embryos. Immunostaining for LPM transcription factors confirmed that forelimb initiation and outgrowth occur while the LPM remains physically continuous. At stage 20/21, the TWIST1-positive somatic LPM formed a unilaminar layer within the broader FOXF1-positive LPM, underlain by a TWIST1-negative, SNAI2-positive splanchnic LPM domain (Fig. 5A). TWIST1-positive cells exhibited a mesenchymal morphology, lacking clear E-cadherin or N-cadherin positive cell-cell junctions and without evident ZO-1-positive apical polarity (Fig. 5B), in contrast to the columnar epithelial organization characteristic of the somatic LPM in chicken or mouse embryos (*18*, *20*). By stages 22/23, the TWIST1-positive limb bud mesenchyme had undergone outgrowth, though remained contiguous with the underlying FOXF1-positive splanchnic LPM (Fig. 5, C and E). Although these domains were dorsoventrally segregated and molecularly distinct, no coelomic cavity was evident between them. By stage 23-25, however, the somatic and splanchnic LPM began to separate, forming the coelomic cavity (Fig. 5, E and G). Together, these observations indicate that limb outgrowth is initiated prior to physical LPM separation, and before condensation of the paraxial mesoderm, which was present as a thin SNAI2-positive layer beneath the neural plate epithelium (Fig. 5, A, C, and E).

To determine whether classical hallmarks of LPM epithelialization and/or rosette formation (*17*) are present prior to or during subdivision, we examined markers of cell adhesion, polarity, and basement membrane organisation. At stages 20-22, the epithelial ectoderm and endoderm showed strong and continuous E-cadherin and laminin staining, whereas the somatic and splanchnic LPM showed weaker and more heterogeneous E- and N-cadherin localization, without clear ZO-1-positive apical enrichment or organised epithelial rosettes (Fig. 5, B and D). By stage 23+ however, these layers progressively resolved into two continuous, columnar, and apically organized coelomic epithelial layers, with aPKC and ZO-1 enrichment at the coelomic boundary and at junctions where separation of the cavity begins (Fig. 5, E and F, and fig. S1). Notably, rather than forming discrete epithelial rosettes, the coelomic boundary appeared to undergo mediolateral separation in a “zipper-like” manner along the anteroposterior axis.

Together, these results indicate that epithelial features within the LPM do not precede forelimb bud outgrowth but instead emerge progressively beneath the expanding limb mesenchyme. Our data are consistent with a model in which molecular subdivision into somatic and splanchnic layers initially occurs within a continuous mesodermal sheet, followed by gradual epithelialisation, polarisation and progressive separation along the forming coelomic boundary. This was seen in place of subdivision via discrete epithelial rosette intermediates, as defined in chicken embryos (*17*). Collectively, these findings highlight the developmental plasticity of the LPM and demonstrate that limb initiation and early outgrowth can occur prior to overt epithelialisation and physical subdivision of the tissue.

### Comparisons of LPM subdivision and forelimb outgrowth across vertebrates

**Fig. 6.**
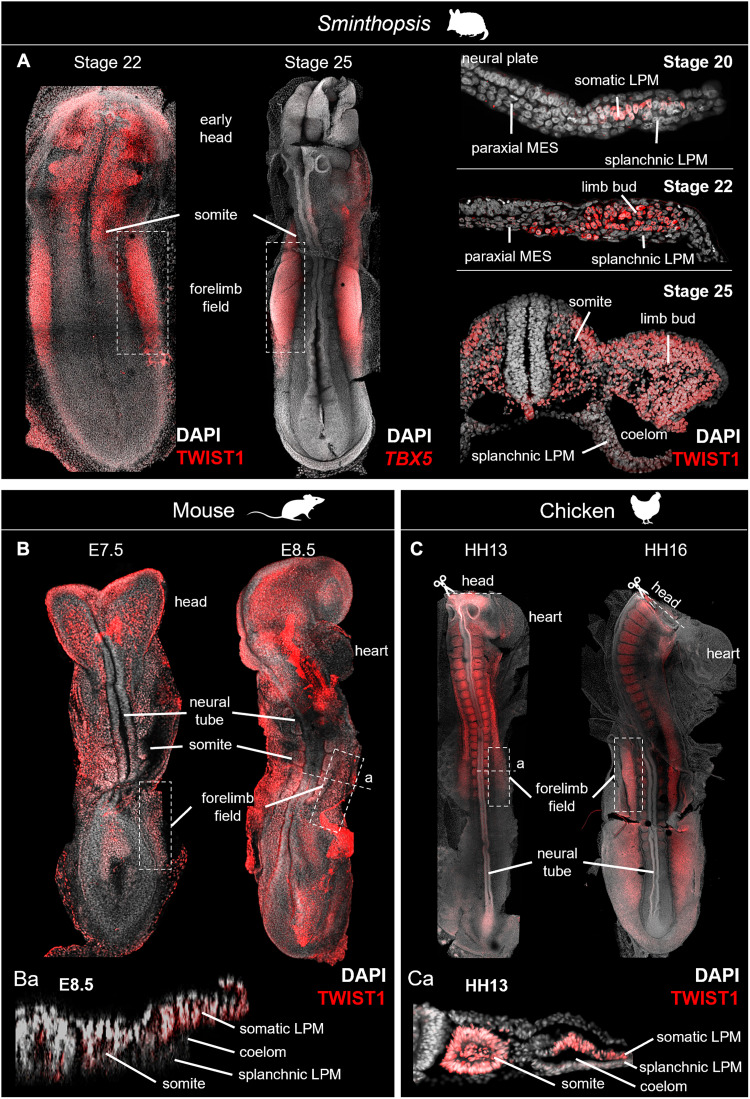
Comparative somatic LPM specification and limb development in model amniotes. Whole mount and tissue section fluorescent imaging showing limb field specification and outgrowth in *Sminthopsis* embryos (**A**), as compared to mouse (**B**) and chicken (**C**) embryos. Stages examined are stated in each panel. Sminthopsis limb specification and early outgrowth occurs between stages 20 and 22, with outgrowth of the limb bud mesenchyme preceding LPM subdivision (A). In mouse embryos, forelimb field specification occurs in early somite stage embryos (E7.5), with initial outgrowth occurring by E8.5 (B). In chicken embryos, limb field specification and outgrowth occur at later somite stages with more advanced embryonic structures (C). In comparison, the marsupial limb fields form at earlier developmental stages lacking other embryonic structures including neural tube closure, condensation of somites and head formation. As such, at relative mouse (E8.5) and chicken (HH14) stages where limb field specification is occurring, *Sminthopsis* embryos already possess distinct forelimb buds. LPM, lateral plate mesoderm; MES, mesoderm.

To better contextualize the morphogenetic differences observed during marsupial limb development with canonical models, we compared TWIST1 immunostaining at the time of forelimb initiation and outgrowth in *Sminthopsis*, mouse and chicken embryos (Fig. 6). In marsupials, forelimb initiation and early outgrowth occur at approximately stage 22, prior to hindlimb specification, somite condensation, neural tube closure, heart looping and expansion of the head prominences (Fig. 6A). In contrast, limb initiation and outgrowth in chicken and mouse embryos occurs at more advanced developmental stages, beginning in the presence of somites, a closed neural tube, and well-established heart and head structures (Fig. 6, B and C). Moreover, forelimb initiation occurs concurrently with [mouse, Fig. 6B (a)], or following [chicken, Fig. 6C (a)] physical subdivision of the LPM. Although the mouse embryo progresses through these stages more rapidly than the chick, the relative sequence of events is conserved.

**Fig. 7.**
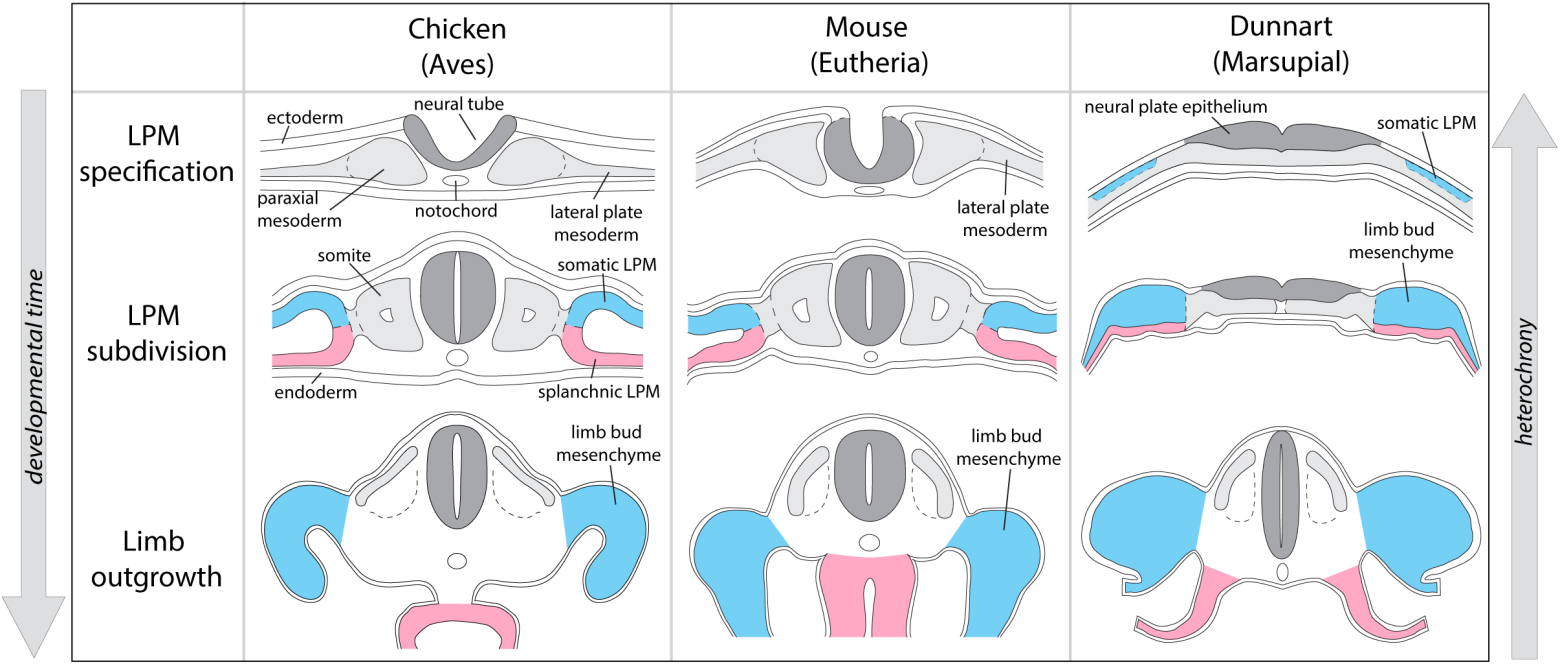
Comparative model of amniote limb development. Marsupials show developmental heterochrony in their LPM specification and limb outgrowth, compared to stage-matched chicken and mouse models.

Marsupials therefore exhibit a distinct temporal shift between limb initiation and these broader developmental landmarks. When marsupial embryos are approximately stage-matched based on axial features, forelimb buds are already densely populated with mesenchyme and visibly protrude from the body wall (Fig. 6). Taken together, these observations demonstrate a pronounced heterochrony in LPM specification and limb outgrowth in marsupials relative to classical tetrapod models (Fig. 7). Rather than indicating a strict uncoupling of these processes, these findings suggest that the temporal coordination between appendicular and axial development can vary substantially across vertebrates. Marsupials initiate limb development well in advance of key organogenic milestones, highlighting flexibility in the relative timing of these developmental programs.

## DISCUSSION

Vertebrate limb development follows a canonical sequence of morphogenetic events within the somatic lateral plate mesoderm (LPM). Following mediolateral segmentation of the mesoderm into axial, paraxial, and lateral domains, the LPM undergoes epithelialization, dorsoventral subdivision into somatic and splanchnic layers, and activation of the TBX5 dependent limb gene regulatory network to initiate EMT and outgrowth of the limb mesenchyme (*16*, *18*, *20*, *23*, *24*, *27*, *45*, *60*–*65*). Although the relative onset of these events varies between species, their generally sequential order has established the prevailing model for limb formation (*11*, *21*, *22*). Here, we show that marsupials deviate from this typical sequence, exhibiting an alternative temporal relationship between morphogenetic events associated with forelimb formation – likely to meet the extreme functional demands for robust forelimbs following their short gestation. While marsupials exhibit precocious activation of the forelimb-associated gene regulatory network (*45*), we further show that this heterochrony extends upstream of TBX5-dependent induction (Fig. 2) and to the level of tissue morphogenesis. In particular, forelimb initiation and early outgrowth occur prior to physical subdivision of the LPM, formation of the coelomic epithelium, and condensation of the somites (Figs. 3 to 5) (*16*, *17*, *20*). Together, these findings indicate that the canonical sequence of morphogenetic events can be temporally reorganised, revealing an unexpected degree of developmental plasticity in the early stages of limb formation.

LPM development and subdivision represent a conserved morphogenetic process across chordates (*11*), including basal lineages such as amphioxus (*66*). In amniotes, recent work has shown that LPM subdivision proceeds as a coordinated anteroposterior and mediolateral wave. The initially mesenchymal LPM undergoes progressive epithelialisation via the formation of intermediate rosettes (*17*), followed by physical separation into somatic and splanchnic layers to become lined by the coelomic epithelia (*16*, *17*, *41*). Then, during forelimb initiation, the somatic LPM undergoes EMT to generate the proliferative limb bud mesenchyme (*20*, *41*). In contrast, our findings suggest that during marsupial forelimb development, the proliferative limb bud mesenchyme is generated from a mesenchymal LPM prior to overt epithelialisation and subdivision of the coelomic epithelium. Importantly, while we detect molecular polarity of the somatic and splanchnic layers, we do not observe evidence for physical subdivision via epithelial rosette intermediates at the level of the forelimb field during outgrowth. Instead, our data are consistent with a model in which LPM subdivision proceeds through progressive epithelialisation and polarisation within a continuous mesodermal sheet, followed by its separation into somatic and splanchnic layers in a manner resembling a mediolateral “zipper-like” opening along the forming coelomic boundary. Together, these data indicate that the limb gene regulatory network can occur prior to the establishment of fully developed epithelial organisation, highlighting its modularity and capacity for temporal uncoupling from canonical morphogenetic processes. In this context, these findings support a model in which the forelimb gene regulatory network operates as a semi-autonomous module within the LPM, whose activation is not strictly dependent on the completion of preceding morphogenetic events.

Our analyses reveal heterochrony and clear contrasts between forelimb and hindlimb development in marsupials. While forelimb initiation occurs prior to physical subdivision of the LPM, hindlimb development appears to follow the canonical sequence observed in other amniotes (*11*, *16*, *20*, *67*). At the level of the presumptive hindlimb field, we show a distinct coelomic cavity is present by stage 24 (Fig. 4), and previous work in *Monodelphis* show that co-expression of hindlimb initiation factors *PITX1* and *TBX4* occurs at stage 26, with expression of *FGF10* and limb bud outgrowth by stage 27 (*45*). Together, these observations demonstrate that, in contrast to the forelimb, hindlimb induction occurs after epithelialisation and physical separation of the LPM. This indicates that the canonical relationship between LPM subdivision and limb initiation is retained in the slower developing hindlimb, with a distinct morphogenetic shift in the accelerated forelimb. This raises important questions about how the forelimb program is initiated under heterochronic conditions, in a physically continuous and incompletely epithelialized LPM. Especially, it remains unclear which inductive signals are necessary and sufficient to activate the forelimb gene regulatory network in advance of the morphogenetic context typically associated with limb initiation, and prior to the full maturation of surrounding axial structures.

TBX5-dependent forelimb initiation is influenced by multiple signaling pathways, including retinoic acid (RA), WNT, and BMP signaling, as well as collinear HOX gene expression (*16*, *18*, *32*, *36*–*41*, *43*). Previous work in *Monodelphis* embryos has shown that *HOXB5* and *HOXC6* exhibit expanded anteroposterior domains, correlating with an enlarged forelimb field spanning eight presumptive somite pairs, as compared to five or six in mouse, chicken, and human embryos (*45*). These spatial shifts in HOX expression may contribute to the expanded and anteriorly displaced forelimb field, yet they do not fully explain the heterochronic advancement of limb induction. Retinoic acid (RA) signaling, in contrast, has been consistently implicated as a key upstream regulator of TBX5 activation and forelimb field specification across vertebrates (*43*, *68*, *69*), with the somites acting as a key signaling centre through the synthesis and supply of RA. However, in marsupial embryos, forelimb initiation and outgrowth occurs before somite condensation, when the paraxial mesoderm exists as a thin, unsegmented sheet beneath the neural plate epithelium (Fig. 5). Despite this reduced structural maturity, the paraxial mesoderm appears capable of supporting components of the RA signaling pathway, as indicated by complementary expression of the RA-synthesis genes *RDH10* and *ALDH1A2* in the LPM and pre-somitic paraxial mesoderm, respectively (Fig. 3). Notably, these expression domains resemble those observed in equivalent stages of mouse and chicken embryos (*43*, *70*–*72*), suggesting that the spatial organisation of RA synthesis is conserved despite differences in morphogenetic context. Moreover, these expression patterns do not directly demonstrate RA signaling activity, and precocious activation of additional factors such as cellular retinoic acid-binding proteins (CRABPs) or RAR/RXR nuclear receptors may be required to contribute to early forelimb induction. Furthermore, contributions from BMP and WNT signaling pathways may also support early somatic LPM specification (*16*, *18*) and TBX5 activation (*43*), although their timing and roles under heterochronic conditions remain untested in marsupial embryos. Nevertheless, the accelerated activation of the marsupial forelimb gene network ahead of other axial structures provides a unique opportunity to dissect the relative contributions and sufficiency of inductive signals underlying forelimb initiation.

Examination of the cellular and molecular events accompanying the accelerated limb development of marsupial embryos offers revised perspectives on the necessary and flexible morphogenetic processes underlying tetrapod limb formation. Our findings show that marsupial forelimb fields are specified in effective isolation from other axial structures, proceeding outside the temporal sequence typically associated with limb development. Here, the marsupial forelimb appears to undergo initiation and outgrowth prior to LPM subdivision and maturation, and without clear evidence that epithelial–mesenchymal transitions are required at this stage. These insights not only provide previously unexplored perspective on the events associated with limb formation but also refine prevailing models of limb development derived from conventional model organisms. By demonstrating that limb field specification can proceed in advance of the coordinated development of surrounding axial structures, marsupials provide a tractable model to dissect the timing, sufficiency, and hierarchy of inductive cues governing forelimb initiation. This work establishes an important foundation for future studies into the core signaling pathways and patterning mechanisms that govern limb field specification prior to activation of the canonical limb program. Marsupials thus emerge as a powerful comparative model for probing evolutionary flexibility within conserved developmental programs and provide unique insights into the fundamental processes underlying vertebrate limb patterning.

## MATERIALS AND METHODS

### Embryo Collection

All animal procedures were conducted in accordance with relevant institutional and national guidelines for the care and use of animals in research. Work involving *S. crassicaudata* was performed from an experimental colony housed within the School of Biosciences at the University of Melbourne, Australia, using procedures approved by the University of Melbourne Animal Ethics Committee (application numbers 10206 & 26864) and carried out in compliance with the Australian Code for the Care and Use of Animals for Scientific Purposes. *Sminthopsis* embryos were obtained using previously described methods (*52*, *73*). *Monodelphis* embryos were sourced from an experimental colony housed within the Department of Molecular, Cell, and Developmental Biology at the University of California, Los Angeles (UCLA). Mouse embryos were collected from an experimental colony housed within the School of Biomedical Sciences at the University of Melbourne, Australia. Chicken embryos were collected from fertilized eggs, sourced from Specialized Breeders Australia (SBA).

Embryos were fixed in 4% paraformaldehyde (PFA) at 4°C overnight. Whole embryos fixed in PFA were washed in RNAse free 1X PBS with 0.1% Tween-20 (PBST) and then dehydrated in a 25%, 50%, 75%, and 100% methanol series (in PBS) on ice. Embryos were then stored in 100% methanol at -20°C until use.

### Wholemount and section fluorescent imaging

Gene expression analysis was performed using immunofluorescence and hybridization chain reaction (HCR) (*74*). Antibodies used in the study are listed in Table 1. HCR probes (Molecular Instrument, Los Angeles, CA) were initially designed against target gene sequences from the dunnart (*S. crassicaudata*) transcriptome (*75*). However, to ensure cross-reactivity between marsupials, probe sequences were BLASTed and retained only if they were specific to the gene of interest and had >85% similarity to *Monodelphis*.

**Table 1.** Antibodies used in the study.

Antibody target	Dilution	Species	Antibody number	Source
TWIST1	1:100	Mouse	AB50887	Abcam
FOXF1	1:100	Goat	AF4798	R&D Systems
SNAIL2	1:200	Rabbit	C19G7	Cell Signaling Technology
E*-*Cadherin	1:200	Mouse	AB76055	Abcam
N-Cadherin	1:200	Mouse	610920	BD Biosciences
Vimentin	1:100	Mouse	AB8069	Abcam
Laminin	1:200	Rabbit	AB11575	Abcam
Zo-1	1:500	Rabbit	40-2200	Thermo Fisher Scientific
p-PKC zeta (aPKC)	1:500	Mouse	sc-271962	Santa Cruz Biotech

For immune labelling of target proteins, dehydrated embryos were transferred directly from 100% methanol into blocking buffer (1X PBS with 0.1% Tween-20 (PBST) with 3% BSA) and left to block for at least 1 hour, rotating at 4°C. Primary antibodies were diluted in PBST with 1% BSA and incubated with embryos overnight at 4°C. On the second day, embryos were washed with PBST, then incubated with secondary antibodies (diluted at 1:500 in PBST with 1% BSA) at room temperature overnight, and counterstained with DAPI (1:10,000 in PBST), before mounting.

HCR was performed using the protocol provided by Molecular Instruments (*74*) with minor modifications based on the stage of the embryos. Briefly, embryos were rehydrated with the reverse methanol series (75%, 50%, 25% in DEPC-PBST) on ice. Embryos between stages 24-25 were incubated with proteinase-K at room temperature for 5 minutes, then post-fixed in DEPC-PFA for 20 minutes at room temperature, while embryos at stages prior to stage 24 were not incubated with proteinase-K. All embryos were incubated with 10-30 pmol of probes for *PRRX1* & *TBX5* in hybridisation buffer at 37°C overnight. On the second day, embryos were washed in wash buffer and DEPC-SSCT and were then incubated with 30 pmol of H1 and H2 hairpins in amplification buffer at room temperature overnight. On the third day, embryos were washed in DEPC-SSCT and incubated with DAPI (1:10,000 in PBST), before being washed again in PBST.

Whole embryos were incubated in 30% sucrose in PBS overnight before embedded in Tissue-Tek O.C.T. mounting media (ProSciTech) and stored at -80 until use. Ten μm cryosections were cut and placed on alternating superfrost slides for successive immunostaining. Sections were washed in 1% Triton-X in 1X PBS (PBTX) and then blocked for at least 1 hour in 2% BSA in PBS at room temperature, before incubated with primary antibodies diluted in PBS at 4°C overnight. Sections were then washed in PBS and incubated with secondary antibodies (1:500 in PBS containing 1% BSA) at room temperature in the dark for 1 hour. Sections were counterstained with DAPI (1:10,000 in PBS), before mounted with ProLong Glass Antifade (Invitrogen) media and cover slipped.

### Imaging and analysis

Embryos and slides were imaged on a Nikon A1R confocal microscope with NIS-Elements software. Whole mount scans were captured with a 10x PL APO Lambda MRD00105 air objective (NA = 0.45), while tissue section images were imaged with a 40x PL FLUO MRH01401 oil objective (NA = 1.3). Due to the size of the wholemount samples, images were taken as large-image Z-stacks and were then stitched together in post-processing to make a single image. All image post-processing was performed using ImageJ (Fiji) for visualization, z-projection or z-slicing.

## References

[1] L. Niswander, Pattern formation: Old models out on a limb. Nat. Rev. Genet. 4, 133–143 (2003). 12560810 10.1038/nrg1001

[2] A. A. Zuniga, Next generation limb development and evolution: Old questions, new perspectives. Development 142, 3810–3820 (2015). 26577204 10.1242/dev.125757

[3] C. Tickle, How the embryo makes a limb: Determination, polarity and identity. J. Anat. 227, 418–430 (2015). 26249743 10.1111/joa.12361 PMC4580101

[4] K. L. Cooper, K. E. Sears, A. Uygur, J. Maier, K. S. Baczkowski, M. Brosnahan, D. Antczak, J. A. Skidmore, C. J. Tabin, Patterning and post-patterning modes of evolutionary digit loss in mammals. Nature 511, 41–45 (2014). 24990742 10.1038/nature13496 PMC4228958

[5] M. A. G. De Bakker, D. A. Fowler, K. Den Oude, E. M. Dondorp, M. C. G. Navas, J. O. Horbanczuk, J. Y. Sire, D. Szczerbińska, M. K. Richardson, Digit loss in archosaur evolution and the interplay between selection and constraints. Nature 500, 445–448 (2013). 23831646 10.1038/nature12336

[6] M. J. Cohn, C. Tickle, Developmental basis of limblessness and axial patterning in snakes. Nature 399, 474–479 (1999). 10365960 10.1038/20944

[7] M. Kmita, B. Tarchini, J. Zàkàny, M. Logan, C. J. Tabin, D. Duboule, Early developmental arrest of mammalian limbs lacking HoxA/HoxD gene function. Nature 435, 1113–1116 (2005). 15973411 10.1038/nature03648

[8] K. Cooper, A. Saxena, V. Sharma, S. Neufeld, M. Tran, H. Gutierrez, J. Erberich, A. Birmingham, J. Cobb, M. Hiller, Interspecies transcriptome analyses identify genes that control the development and evolution of limb skeletal proportion. FASEB J. 34, 1 (2020).

[9] J. Lopez-Rios, A. Duchesne, D. Speziale, G. Andrey, K. A. Peterson, P. Germann, E. Ünal, J. Liu, S. Floriot, S. Barbey, Y. Gallard, M. Müller-Gerbl, A. D. Courtney, C. Klopp, S. Rodriguez, R. Ivanek, C. Beisel, C. Wicking, D. Iber, B. Robert, A. P. McMahon, D. Duboule, R. Zeller, Attenuated sensing of SHH by Ptch1 underlies evolution of bovine limbs. Nature 511, 46–51 (2014). 24990743 10.1038/nature13289

[10] F. Petit, K. E. Sears, N. Ahituv, Limb development: A paradigm of gene regulation. Nat. Rev. Genet. 18, 245–258 (2017). 28163321 10.1038/nrg.2016.167

[11] K. D. Prummel, S. Nieuwenhuize, C. Mosimann, The lateral plate mesoderm. Development 147, dev175059 (2020). 32561665 10.1242/dev.175059 PMC7328003

[12] D. Sela-Donenfeld, C. Kalcheim, Localized BMP4-noggin interactions generate the dynamic patterning of noggin expression in somites. Dev. Biol. 246, 311–328 (2002). 12051818 10.1006/dbio.2002.0672

[13] A. Rojas, S. De Val, A. B. Heidt, S. M. Xu, J. Bristow, B. L. Black, Gata4 expression in lateral mesoderm is downstream of BMP4 and is activated directly by Forkhead and GATA transcription factors through a distal enhancer element. Development 132, 3405–3417 (2005). 15987774 10.1242/dev.01913

[14] C. D. Tsiairis, A. P. Mcmahon, An Hh-Dependent Pathway in Lateral Plate Mesoderm Enables the Generation of Left/Right Asymmetry. Curr. Biol. 19, 1912–1917 (2009). 19879143 10.1016/j.cub.2009.09.057 PMC2787870

[15] A. Tonegawa, Y. Takahashi, Somitogenesis Controlled by Noggin. Dev. Biol. 202, 172–182 (1998). 9769170 10.1006/dbio.1998.8895

[16] N. Funayama, Y. Sato, K. Matsumoto, T. Ogura, Y. Takahashi, Coelom formation: Binary decision of the lateral plate mesoderm is controlled by the ectoderm. Development 126, 4129–4138 (1999). 10457021 10.1242/dev.126.18.4129

[17] M. Abboud Asleh, M. Zaher, J. Asleh, J. Jadon, L. Shaulov, R. Yelin, T. M. Schultheiss, A morphogenetic wave in the chick embryo lateral mesoderm generates mesenchymal-epithelial transition through a 3D-rosette intermediate. Dev. Cell 58, 951–966.e5 (2023). 37080204 10.1016/j.devcel.2023.03.017

[18] A. H. Newton, S. M. Williams, A. T. Major, C. A. Smith, Cell lineage specification and signalling pathway use during development of the lateral plate mesoderm and forelimb mesenchyme. Development 149, dev200702 (2022). 36093878 10.1242/dev.200702

[19] S. Nishimoto, M. P. O. Logan, Subdivision of the lateral plate mesoderm and specification of the forelimb and hindlimb forming domains. Semin. Cell Dev. Biol. 49, 102–108 (2016). 26643124 10.1016/j.semcdb.2015.11.011

[20] J. Gros, C. J. Tabin, Vertebrate Limb Bud Formation Is Initiated by Localized Epithelial-to-Mesenchymal Transition. Science 343, 1253–1256 (2014). 24626928 10.1126/science.1248228 PMC4097009

[21] M. Tanaka, Molecular and evolutionary basis of limb field specification and limb initiation. Dev. Growth Differ. 55, 149–163 (2013). 23216351 10.1111/dgd.12017

[22] M. Tanaka, Developmental Mechanism of Limb Field Specification along the Anterior–Posterior Axis during Vertebrate Evolution. J. Dev. Biol. 4, 18 (2016). 29615584 10.3390/jdb4020018 PMC5831784

[23] S. Kuratani, J. F. Martin, S. Wawersik, B. Lilly, G. Eichele, E. N. Olson, The expression pattern of the chick homeobox gene gMHox suggests a role in patterning of the limbs and face and in compartmentalization of somites. Dev. Biol. 161, 357–369 (1994). 7906232 10.1006/dbio.1994.1037

[24] B. Leussink, A. Brouwer, M. El Khattabi, R. E. Poelmann, A. C. Gittenberger-de Groot, F. Meijlink, Expression patterns of the paired-related homeobox genes MHox Prx1 and S8 Prx2 suggest roles in development of the heart and the forebrain. Mech. Dev. 52, 51–64 (1995). 7577675 10.1016/0925-4773(95)00389-i

[25] A. T. Tavares, J. C. Izpisúa-Belmonte, J. Rodríguez-León, Developmental expression of chick Twist and its regulation during limb patterning. Int. J. Dev. Biol. 45, 707–713 (2001). 11669372

[26] I. Gitelman, Twist protein in mouse embryogenesis. Dev. Biol. 189, 205–214 (1997). 9299114 10.1006/dbio.1997.8614

[27] M. Mahlapuu, M. Ormestad, S. Enerbäck, P. Carlsson, The forkhead transcription factor Foxf1 is required for differentiation of extra-embryonic and lateral plate mesoderm. Development 128, 155–166 (2001). 11124112 10.1242/dev.128.2.155

[28] Z. F. Chen, R. R. Behringer, Twist Is Required in Head Mesenchyme for Cranial Neural Tube Morphogenesis. Genes Dev. 9, 686–699 (1995). 7729687 10.1101/gad.9.6.686

[29] D. ten Berge, A. Brouwer, J. Korving, J. F. Martin, F. Meijlink, Prx1 and Prx2 in skeletogenesis: roles in the craniofacial region, inner ear and limbs. Development 125, 3831–3842 (1998). 9729491 10.1242/dev.125.19.3831

[30] P. Agarwal, J. N. Wylie, J. Galceran, O. Arkhitko, C. Li, C. Deng, R. Grosschedl, B. G. Bruneau, Tbx5 is essential for forelimb bud initiation following patterning of the limb field in the mouse embryo. Development 130, 623–633 (2003). 12490567 10.1242/dev.00191

[31] C. Rallis, B. G. Bruneau, J. Del Buono, C. E. Seidman, J. G. Seidman, S. Nissim, C. J. Tabin, M. P. O. Logan, Tbx5 is required for forelimb bud formation and continued outgrowth. Development 130, 2741–2751 (2003). 12736217 10.1242/dev.00473

[32] J. K. Ng, Y. Kawakami, D. Büscher, Á. Raya, T. Itoh, C. M. Koth, C. R. Esteban, J. Rodríguez-León, D. M. Garrity, M. C. Fishman, J. Carlos, I. Belmonte, C. Rodríguez Esteban, J. Rodríguez-León, D. M. Garrity, M. C. Fishman, J. C. I. Belmonte, The limb gene Tbx5 promotes limb initiation by interacting with Wnt2b and Fgf10. Development 129, 5161–5170 (2002). 12399308 10.1242/dev.129.22.5161

[33] K. Sekine, H. Ohuchi, M. Fujiwara, M. Yamasaki, T. Yoshizawa, T. Sato, N. Yagishita, D. Matsui, Y. Koga, N. Itoh, S. Kato, Fgf10 is essential for limb and lung formation. Nat. Genet. 21, 138–141 (1999). 9916808 10.1038/5096

[34] H. Ohuchi, T. Nakagawa, A. Yamamoto, A. Araga, T. Ohata, Y. Ishimaru, H. Yoshioka, T. Kuwana, T. Nohno, M. Yamasaki, N. Itoh, S. Noji, The mesenchymal factor, FGF10, initiates and maintains the outgrowth of the chick limb bud through interaction with FGF8, an apical ectodermal factor. Development 124, 2235–2244 (1997). 9187149 10.1242/dev.124.11.2235

[35] A. M. Moon, M. R. Capecchi, Fgf8 is required for outgrowth and patterning of the limbs. Nat. Genet. 26, 455–459 (2000). 11101845 10.1038/82601 PMC2001274

[36] C. Minguillon, S. Nishimoto, S. Wood, E. Vendrell, J. J. Gibson-Brown, M. P. O. Logan, Hox genes regulate the onset of Tbx5 expression in the forelimb. Dev. 139, 3180–3188 (2012). 10.1242/dev.084814 PMC3413163 22872086

[37] C. Moreau, P. Caldarelli, D. Rocancourt, J. Roussel, N. Denans, O. Pourquie, J. Gros, Timed Collinear Activation of Hox Genes during Gastrulation Controls the Avian Forelimb Position. Curr. Biol. 29, 35–50.e4 (2019). 30554902 10.1016/j.cub.2018.11.009 PMC6331352

[38] M. J. Cohn, K. Patel, R. Krumlauf, D. G. Wilkinson, J. D. W. Clarke, C. Tickle, Hox9 genes and vertebrate limb specification. Nature 387, 97–100 (1997). 9139829 10.1038/387097a0

[39] S. Nishimoto, C. Minguillon, S. Wood, M. P. O. Logan, A Combination of Activation and Repression by a Colinear Hox Code Controls Forelimb-Restricted Expression of Tbx5 and Reveals Hox Protein Specificity. PLOS Genet. 10, e1004245 (2014). 24651482 10.1371/journal.pgen.1004245 PMC3961185

[40] Y. Kawakami, J. Capdevila, D. Büscher, T. Itoh, C. R. Esteban, J. C. I. Belmonte, WNT signals control FGF-dependent limb initiation and AER induction in the chick embryo. Cell 104, 891–900 (2001). 11290326 10.1016/s0092-8674(01)00285-9

[41] A. H. Newton, C. A. Smith, Resolving the mechanisms underlying epithelial-to-mesenchymal transition of the lateral plate mesoderm. Genesis 62, e23531 (2023). 37443419 10.1002/dvg.23531

[42] J. S. Waxman, B. R. Keegan, R. W. Roberts, K. D. Poss, D. Yelon, Hoxb5b Acts Downstream of Retinoic Acid Signaling in the Forelimb Field to Restrict Heart Field Potential in Zebrafish. Dev. Cell 15, 923–934 (2008). 19081079 10.1016/j.devcel.2008.09.009 PMC2752051

[43] S. Nishimoto, S. M. Wilde, S. Wood, M. P. O. Logan, RA Acts in a Coherent Feed-Forward Mechanism with Tbx5 to Control Limb Bud Induction and Initiation. Cell Rep. 12, 879–891 (2015). 26212321 10.1016/j.celrep.2015.06.068 PMC4553633

[44] T. Stratford, C. Horton, M. Maden, Retinoic acid is required for the initiation of outgrowth in the chick limb bud. Curr. Biol. 6, 1124–1133 (1996). 8805369 10.1016/s0960-9822(02)70679-9

[45] A. L. Keyte, K. K. Smith, Developmental origins of precocial forelimbs in marsupial neonates. Development 137, 4283–4294 (2010). 21098569 10.1242/dev.049445

[46] K. K. Smith, A. L. Keyte, Adaptations of the Marsupial Newborn: Birth as an Extreme Environment. Anat. Rec. 303, 235–249 (2020). 10.1002/ar.24049 30548826

[47] L. E. Cook, A. H. Newton, C. A. Hipsley, A. J. Pask, Postnatal development in a marsupial model, the fat-tailed dunnart (Sminthopsis crassicaudata; Dasyuromorphia: Dasyuridae). Commun. Biol. 4, 1028 (2021). 34475507 10.1038/s42003-021-02506-2 PMC8413461

[48] A. Keyte, K. K. Smith, Heterochrony in somitogenesis rate in a model marsupial, *Monodelphis domestica*. Evol. Dev. 14, 93–103 (2012). 23016977 10.1111/j.1525-142X.2011.00524.x

[49] C. K. Doroba, K. E. Sears, The divergent development of the apical ectodermal ridge in the marsupial Monodelphis domestica. Anat. Rec. 293, 1325–1332 (2010). 10.1002/ar.21183 20665811

[50] K. Y. Chew, H. Yu, A. J. Pask, G. Shaw, M. B. Renfree, HOXA13 and HOXD13 expression during development of the syndactylous digits in the marsupial Macropus eugenii. BMC Dev. Biol. 12, 2 (2012). 22235805 10.1186/1471-213X-12-2 PMC3268106

[51] K. Y. Chew, G. Shaw, H. Yu, A. J. Pask, M. B. Renfree, Heterochrony in the regulation of the developing marsupial limb. Dev. Dyn. 243, 324–338 (2014). 24115631 10.1002/dvdy.24062

[52] A. H. Newton, J. C. Hutchison, E. R. Farley, E. L. Scicluna, N. A. Youngson, J. Liu, B. R. Menzies, T. B. Hildebrandt, B. M. Lawrence, A. H. W. Sutherland, D. L. Potter, G. A. Tarulli, L. Selwood, S. Frankenberg, S. Ord, A. J. Pask, Embryology of the fat-tailed dunnart (Sminthopsis crassicaudata): A marsupial model for comparative mammalian developmental and evolutionary biology. Dev. Dyn. 254, 142–157 (2024). 38721717 10.1002/dvdy.711 PMC11809135

[53] O. R. P. Bininda-Emonds, M. Cardillo, K. E. Jones, R. D. E. MacPhee, R. M. D. Beck, R. Grenyer, S. A. Price, R. A. Vos, J. L. Gittleman, A. Purvis, The delayed rise of present-day mammals. Nature 446, 507–512 (2007). 17392779 10.1038/nature05634

[54] K. E. Mate, E. S. Robinson, R. A. Pedersen, J. L. Vandeberg, Timetable of in vivo embryonic development in the grey short-tailed opossum (Monodelphis domestica). Mol. Reprod. Dev. 39, 365–374 (1994). 7893485 10.1002/mrd.1080390404

[55] J. F. Martin, E. N. Olson, Identification of a prx1 limb enhancer. Genesis 26, 225–229 (2000). 10748458

[56] M. Logan, J. F. Martin, A. Nagy, C. Lobe, E. N. Olson, C. J. Tabin, Expression of Cre Recombinase in the developing mouse limb bud driven by a Prxl enhancer. Genesis 33, 77–80 (2002). 12112875 10.1002/gene.10092

[57] J. F. Martin, A. Bradley, E. N. Olson, N. O. Eric, The paired-like homeo box gene MHox is required for early events of skeletogenesis in multiple lineages. Genes Dev. 9, 1237–1249 (1995). 7758948 10.1101/gad.9.10.1237

[58] C. Rodriguez-Esteban, T. Tsukui, S. Yonei, J. Magallon, K. Tamura, J. C. Izpisua Belmonte, The T-box genes Tbx4 and Tbx5 regulate limb outgrowth and identity. Nature 398, 814–818 (1999). 10235264 10.1038/19769

[59] J. K. Takeuchi, K. Koshiba-Takeuchi, K. Matsumoto, A. Vogel-Höpker, M. Naitoh-Matsuo, K. Ogura, N. Takahashi, K. Yasuda, T. Ogura, Tbx5 and Tbx4 genes determine the wing/leg identity of limb buds. Nature 398, 810–814 (1999). 10235263 10.1038/19762

[60] E. L. Green, *Biology of the Laboratory Mouse / by the Staff of the Jackson Laboratory* (Dover Publications, New York, 2d ed., 1966; https://books.google.pl/books?id=kqLKcQAACAAJ).

[61] K. Theiler, *The House Mouse* (Springer Berlin Heidelberg, Berlin, Heidelberg, Heidelberg, 1989; 10.1007/978-3-642-88418-4)vol) vol. 7.

[62] V. Hamburger, H. L. Hamilton, A series of normal stages in the development of the chick embryo. J. Morphol. 88, 49–92 (1951). 24539719

[63] K. Soo, M. P. O’Rourke, P. L. Khoo, K. A. Steiner, N. Wong, R. R. Behringer, P. P. L. Tam, Twist function is required for the morphogenesis of the cephalic neural tube and the differentiation of the cranial neural crest cells in the mouse embryo. Dev. Biol. 247, 251–270 (2002). 12086465 10.1006/dbio.2002.0699

[64] E. M. Füchtbauer, Expression of M-twist during postimplantation development of the mouse. Dev. Dyn. 204, 316–322 (1995). 8573722 10.1002/aja.1002040309

[65] D.-J. E. J. E. Opstelten, R. Vogels, B. Robert, E. Kalkhoven, F. Zwartkruis, L. de Laaf, O. H. Destrée, J. Deschamps, K. A. Lawson, F. Meijlink, The mouse homeobox gene, S8, is expressed during embryogenesis predominantly in mesenchyme. Mech. Dev. 34, 29–41 (1991). 1680375 10.1016/0925-4773(91)90089-o

[66] K. Onimaru, E. Shoguchi, S. Kuratani, M. Tanaka, Development and evolution of the lateral plate mesoderm: Comparative analysis of amphioxus and lamprey with implications for the acquisition of paired fins. Dev. Biol. 359, 124–136 (2011). 21864524 10.1016/j.ydbio.2011.08.003

[67] K. D. Prummel, C. Hess, S. Nieuwenhuize, H. J. Parker, K. W. Rogers, I. Kozmikova, C. Racioppi, E. C. Brombacher, A. Czarkwiani, D. Knapp, S. Burger, E. Chiavacci, G. Shah, A. Burger, J. Huisken, M. H. Yun, L. Christiaen, Z. Kozmik, P. Müller, M. Bronner, R. Krumlauf, C. Mosimann, A conserved regulatory program initiates lateral plate mesoderm emergence across chordates. Nat. Commun. 10, 3857 (2019). 31451684 10.1038/s41467-019-11561-7 PMC6710290

[68] Y. Gibert, A. Gajewski, A. Meyer, G. Begemann, Induction and prepatterning of the zebrafish pectoral fin bud requires axial retinoic acid signaling. Development 133, 2649–2659 (2006). 16774994 10.1242/dev.02438

[69] X. Zhao, I. O. Sirbu, F. A. Mic, N. Molotkova, A. Molotkov, S. Kumar, G. Duester, Retinoic Acid Promotes Limb Induction through Effects on Body Axis Extension but Is Unnecessary for Limb Patterning. Curr. Biol. 19, 1050–1057 (2009). 19464179 10.1016/j.cub.2009.04.059 PMC2701469

[70] T. J. Cunningham, X. Zhao, L. L. Sandell, S. M. Evans, P. A. Trainor, G. Duester, Antagonism between Retinoic Acid and Fibroblast Growth Factor Signaling during Limb Development. Cell Rep. 3, 1503–1511 (2013). 23623500 10.1016/j.celrep.2013.03.036 PMC3745640

[71] T. Hochgreb, V. L. Linhares, D. C. Menezes, A. C. Sampaio, C. Y. I. Yan, W. V. Cardoso, N. Rosenthal, J. Xavier-Neto, A caudorostral wave of RALDH2 conveys anteroposterior information to the cardiac field. Development 130, 5363–5374 (2003). 13129847 10.1242/dev.00750

[72] L. L. Sandell, B. W. Sanderson, G. Moiseyev, T. Johnson, A. Mushegian, K. Young, J. P. Rey, J. X. Ma, K. Staehling-Hampton, P. A. Trainor, RDH10 is essential for synthesis of embryonic retinoic acid and is required for limb, craniofacial, and organ development. Genes Dev. 21, 1113–1124 (2007). 17473173 10.1101/gad.1533407 PMC1855236

[73] E. L. Scicluna, *Sminthopsis crassicaudata* colony best practices. Dev. Dyn. 254, 189–204 (2024). 10.1002/dvdy.755 PMC11809136 39895010

[74] H. M. T. T. Choi, M. Schwarzkopf, M. E. Fornace, A. Acharya, G. Artavanis, J. Stegmaier, A. Cunha, N. A. Pierce, Third-generation in situ hybridization chain reaction: Multiplexed, quantitative, sensitive, versatile, robust. Development 145, dev165753 (2018). 29945988 10.1242/dev.165753 PMC6031405

[75] N. Ibeh, C. Y. Feigin, S. R. Frankenberg, J. Davis, A. J. Pask, I. G. Romero, De novo transcriptome assembly and genome annotation of the fat-tailed dunnart (Sminthopsis crassicaudata). bioRxiv, 10.1101/2023.11.16.567318 (2023). 10.46471/gigabyte.118 PMC11091235 38746537

