# Supplementary material for: Heterochronic limb patterning in marsupials reveals flexibility in the processes underlying lateral plate mesoderm morphogenesis
Source: Sci Adv. 2026 Jul 15;12(29):eaed3192. doi: 10.1126/sciadv.aed3192 (PMC13371925; doi:10.1126/sciadv.aed3192)
Supplement: Supplementary file 1 — Fig. S1 [file sciadv.aed3192_sm.pdf]

Science Advances
AAAS

Supplementary Materials for

Heterochronic limb patterning in marsupials reveals flexibility in the processes underlying lateral plate mesoderm morphogenesis

Axel H Newton *et al.*

Corresponding author: Axel H Newton, axel.newton@unimelb.edu.au

Sci. Adv. **12**, eaed3192 (2026)
DOI: 10.1126/sciadv.aed3192

This PDF file includes:

Fig. S1

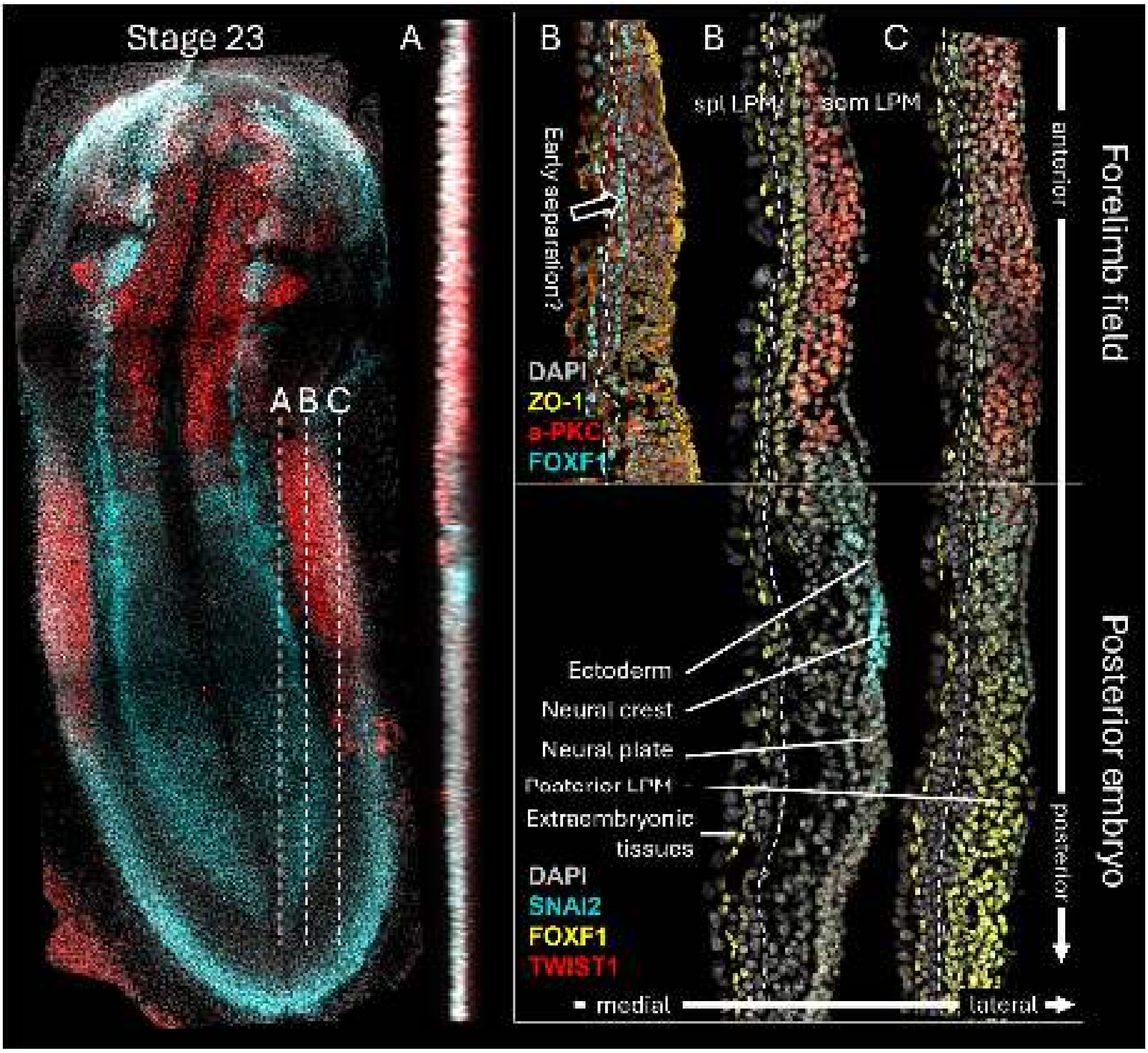

Supplementary Figure 1 – Longitudinal sections of the stage 23 embryo show molecular subdivision along the anteroposterior axis. Wholemount and A) optical longitudinal section of the stage 23 *Sminthopsis* embryo, stained for TWIST1 and SNAI2 showing the TWIST1 positive forelimb field. High resolution, longitudinal tissue section immunostained for B) cell polarity markers aPKC and ZO-1 (B) and TWIST1, FOXF1 and SNAI2 to show the somatic LPM, splanchnic LPM and non-LPM tissues, respectively. Similar to Figure 5, TWIST1-positive somatic LPM and FOXF1-positive splanchnic LPM domains are molecularly segregated but remain physically continuous, albeit with some evidence of aPKC enriched physical subdivision occurring at the medial edge of the forelimb field (black arrow). Note, additional extraembryonic tissues are attached to ventral side of the embryo (dashed line), particularly observed by appearance of large, round nuclei corresponding to the extraembryonic ectoderm.
